# Hardwood Tree Genomics: Unlocking Woody Plant Biology

**DOI:** 10.3389/fpls.2018.01799

**Published:** 2018-12-17

**Authors:** Gerald A. Tuskan, Andrew T. Groover, Jeremy Schmutz, Stephen Paul DiFazio, Alexander Myburg, Dario Grattapaglia, Lawrence B. Smart, Tongming Yin, Jean-Marc Aury, Antoine Kremer, Thibault Leroy, Gregoire Le Provost, Christophe Plomion, John E. Carlson, Jennifer Randall, Jared Westbrook, Jane Grimwood, Wellington Muchero, Daniel Jacobson, Joshua K. Michener

**Affiliations:** ^1^Center for Bioenergy Innovation, Biosciences Division, Oak Ridge National Laboratory (DOE), Oak Ridge, TN, United States; ^2^Pacific Southwest Research Station, USDA Forest Service, Davis, CA, United States; ^3^HudsonAlpha Institute for Biotechnology, Huntsville, AL, United States; ^4^Joint Genome Institute, Walnut Creek, CA, United States; ^5^Department of Biology, West Virginia University, Morgantown, WV, United States; ^6^Department of Biochemistry, Genetics and Microbiology, Forestry and Agricultural Biotechnology Institute, University of Pretoria, Pretoria, South Africa; ^7^Embrapa Recursos Genéticos e Biotecnologia, Brasília, Brazil; ^8^Universidade Católica de Brasília, Brasília, Brazil; ^9^Horticulture Section, School of Integrative Plant Science, Cornell University, Geneva, NY, United States; ^10^The Key Laboratory for Poplar Improvement of Jiangsu Province, Nanjing Forestry University, Nanjing, China; ^11^Commissariat à l’Energie Atomique, Genoscope, Institut de Biologie François-Jacob, Evry, France; ^12^BIOGECO, INRA, Université de Bordeaux, Cestas, France; ^13^ISEM, CNRS, IRD, EPHE, Université de Montpellier, Montpellier, France; ^14^Schatz Center for Tree Molecular Genetics, Department of Ecosystem Science and Management, Pennsylvania State University, University Park, PA, United States; ^15^Department of Entomology, Plant Pathology and Weed Science, New Mexico State University, Las Cruces, NM, United States; ^16^The American Chestnut Foundation, Asheville, NC, United States

**Keywords:** tree habit, somatic mutations, evolutionary ecology, quantitative genetics, adaptive traits, comparative genomics

## Abstract

Woody perennial angiosperms (i.e., hardwood trees) are polyphyletic in origin and occur in most angiosperm orders. Despite their independent origins, hardwoods have shared physiological, anatomical, and life history traits distinct from their herbaceous relatives. New high-throughput DNA sequencing platforms have provided access to numerous woody plant genomes beyond the early reference genomes of *Populus* and *Eucalyptus*, references that now include willow and oak, with pecan and chestnut soon to follow. Genomic studies within these diverse and undomesticated species have successfully linked genes to ecological, physiological, and developmental traits directly. Moreover, comparative genomic approaches are providing insights into speciation events while large-scale DNA resequencing of native collections is identifying population-level genetic diversity responsible for variation in key woody plant biology across and within species. Current research is focused on developing genomic prediction models for breeding, defining speciation and local adaptation, detecting and characterizing somatic mutations, revealing the mechanisms of gender determination and flowering, and application of systems biology approaches to model complex regulatory networks underlying quantitative traits. Emerging technologies such as single-molecule, long-read sequencing is being employed as additional woody plant species, and genotypes within species, are sequenced, thus enabling a comparative (“evo-devo”) approach to understanding the unique biology of large woody plants. Resource availability, current genomic and genetic applications, new discoveries and predicted future developments are illustrated and discussed for poplar, eucalyptus, willow, oak, chestnut, and pecan.

## Introduction

Forest trees have historically been classified as softwood or hardwoods for practical purposes. Botanically, softwood trees are gymnosperms and hardwood trees are angiosperms (i.e., flowering plants) and, as the names indicate, there are differences in reproductive biology and overall habit between these two groups. Around the globe, hardwood trees underpin complex ecosystems, sequester carbon (e.g., rainforests), provide raw materials for the forest products industry, are a source of primary energy in developing countries and are increasingly utilized as a source of renewable bioenergy, biomaterials, and other bioproducts (Ragauskas et al., 2014; FAO, 2016).

Woody perennial angiosperms, i.e., hardwood trees, are amazingly diverse in terms of morphology, life habits, physiology, among many other traits. Hardwood trees show extensive variation in wood anatomy, leaf morphology, whole-tree architecture, secondary metabolism and numerous adaptive traits (Groover and Crook, 2017). The classification of “tree” or arboreal form reflects several distinctive features: e.g., extensive wood formation produced by a vascular or secondary cambium, persistent interannual growth, single or multiple stems exceeding two meters in height, accompanied by perennial lateral branches. Individually, these defining features can however occur outside of the classification as a tree, e.g., some annuals that can produce woody tissue or achieve heights greater than 2 m. Exception aside, hardwood trees can establish extensive root systems that enable vigorous interannual growth, store energy and nutrients overwinter in stem and root tissues, and in many scenarios, create dominant ecosystem overstories.

The ancestral habit for angiosperms is believed to be reflected in the most basal extant angiosperm, *Amborella trichopoda* (Soltis et al., 2008; Albert et al., 2013), which is a small tree native to New Caledonia. During angiosperm evolution, woody perennial habit has been accentuated in some lineages, lost in others (e.g., monocots), and lost and then regained in still others (Spicer and Groover, 2010). To a large extent, these evolutionary and developmental changes are contained in the genomes of the extant hardwood tree species.

Reflecting their evolutionary history, hardwood trees genomes display divergent chromosomal architectures, with multiple ancestral whole-genome duplications and rearrangements, gene family expansions, and many segmental deletions across lineages (Tuskan et al., 2006; Soltis et al., 2008; Dai et al., 2014; Myburg et al., 2014; Plomion et al., 2018). Understanding comparative connections among genomes and the associated characteristics defining hardwood trees remains a substantial research challenge. In this review we will cover the current state of the science in five major groups of hardwood trees outlining the current assembly and annotation metrics, as well as presenting on-going application of these resources. We will highlight on-line resources for these genomes and provide comparative resources for other angiosperm species. We will provide projections of near-term technologies and analytics that will increase the availability and research value of genomes of hardwood trees and conclude with a summary of several unanswered questions related to macro- and micro-evolution of woody perennials that can be addressed using genomic approaches in future.

## State of the Science

### Populus

When the *Populus trichocarpa* genome, ‘Nisquallly-1,’ was released in 2006 it was the first plant genome to use shotgun sequencing assembly approaches, the first genome to create chromosome-level assemblies based on genetic mapping, the first woody perennial genome to be assembled and annotated, as well as the first metagenomic assembly of associated endophytic bacteria and fungi (Tuskan et al., 2006). Today, v3.2 contains 423 Mb [of the ∼485 Mb genome] in 1,446 scaffolds with 2,585 gaps^1^. The scaffold N50 metric is 8 Mb and contig N50 is 205 kb, with roughly 98% of the genome in 181 scaffolds. Trained on deep RNAseq data from the Gene Atlas project^2^, there are 41,335 predicted loci and 73,013 protein coding transcripts. Methylation maps are available for 10 tissue types, including bud tissue, callus tissue, female catkins, internode explants, male catkins, phloem, xylem, and roots (Vining et al., 2012). Over 28 million single nucleotide polymorphisms (SNPs) from resequenced data representing over 880 native *P*. *trichocarpa* genotypes covering the core distribution of the species range are publicly available (Geraldes et al., 2013^3^). This SNP resource has been used to characterize geographic structure and linkage disequilibrium (Slavov et al., 2012), detect signatures of selection across the genome (Evans et al., 2014) and identify genetic loci associated with various phenotypes based on genome-wide association approaches (Porth et al., 2013; Evans et al., 2014; McKown et al., 2014; Muchero et al., 2015; Fahrenkrog et al., 2017; Liu et al., 2018). The resequenced data is currently being assembled into a *Populus* pan genome, with preliminary data suggesting that there may be as many as 20,000 additional gene models that are not included in the current *P. trichocarpa* reference genome annotation (Pinosio et al., 2016). The first draft assemblies of *Populus deltoides*, ‘WV94’ and *Populus tremula* ×*alba* hybrid, ‘717-1B4,’ have been sequenced, assembled and annotated^1^. The assembly for WV94 is approximately 446 Mb in 1,375 scaffolds, with scaffold N50 of 21.7 Mb and contig N50 of 590 kb and there are 44,853 protein coding loci. CRISPR-related PAM (protospacer adjacent motif) sites for *P. tremula* ×*alba* 717 have been published are on line at http://aspendb.uga.edu/s717. Highly contiguous, *de novo* genome assemblies have also been produced for *P*. *euphratica* (Ma et al., 2014) and *P*. *tremula*^4^, which greatly widen the phylogenetic sampling of the genus. Ongoing resource development work in *Populus* includes efforts to expand the tissue type and experimental conditions in the Gene Atlas, enlarge the number resequenced genotypes from the southern and eastern portions of the range, and release of V4.0 of the *P*. *trichocarpa* genome based on long-read sequencing and dense genetic maps derived from resequencing 1000 progeny from 49 half-sib families. Dozens of genome-wide association studies continue across a broad array of phenotypes and efforts to estimate the somatic mutation rate found in old-growth *P*. *trichocarpa* are currently underway. Methods to integrate SNP, gene expression, transcription factor binding, gene dosage, methylation, metabolite expression, phenome, and co-evolutionary relationships have been developed to generate a systems biology view of the molecular and regulatory interactions that lead to organismal scale traits (Henry et al., 2015; Liu et al., 2015; Joubert et al., 2017, 2018; Weighill et al., 2018). New algorithms are being developed to use signal processing techniques as well as explainable artificial intelligence to build better systems biology models, which include methods to discover and model genome-wide epistasis and pleiotropy. Efforts are also underway to gain a comprehensive view of chromatin structure and transcription factor binding sites (Rao et al., 2014).

### Eucalyptus

*Eucalyptus* species and hybrids represent the most widely cultivated hardwood biomass crop globally and *Eucalyptus grandis* (rose gum) clonal genotype ‘BRASUZ1,’ was selected as the reference for the genus. Published in 2014, the *E. grandis* genome was the first for the rosid order Myrtales and for the family Myrtaceae. Key features of the genome included a very high proportion (34%) and number of genes (over 12,500) in tandem duplicate arrays, an ancient (109 Mya) genome-wide duplication event and high diversity of gene families encoding plant specialized metabolites such as phenylpropanoids and terpenes important for plant defense and pharmaceutical uses such as eucalyptus oils (Mewalal et al., 2017). BRASUZ1 was one of the last plant genomes produced exclusively with Sanger technology combined with extensive BAC-end coverage, resulting in a high-quality assembly (currently V2.0^1^) comprising 691 Mb in 4,943 scaffolds with a scaffold N50 of 57.5 Mb, contig N50 of 67 kb and 94% of the estimated 640 Mb genome (Grattapaglia and Bradshaw, 1994) in 11 pseudomolecules containing 288 scaffolds longer than 50 kb. A total of 36,349 protein coding loci are annotated and the assembly has served as reference for extensive transcriptomics studies targeting the regulation and biosynthesis of lignocellulosic biomass (Mizrachi et al., 2014; Carocha et al., 2015; Hussey et al., 2015; Soler et al., 2015) and in comparative analyses with *Arabidopsis* (Davin et al., 2016) and *Populus* (Hefer et al., 2015; Pinard et al., 2015). Other research targets have included terpene biosynthesis (Myburg et al., 2014; Kulheim et al., 2015), reproductive biology (Vining et al., 2015), plant defense (Christie et al., 2015; Mangwanda et al., 2015; Oates et al., 2015; Meyer et al., 2016) and abiotic interactions (Plett et al., 2015; Spokevicius et al., 2017). High-throughput transcriptome sequencing in 100s of segregating interspecific hybrids of *E. grandis* ×*E. urophylla* have been used to perform the first comprehensive systems genetics analysis of wood development in *Eucalyptus* (Mizrachi et al., 2017). The *E. grandis* genome has also served as a reference for whole-genome analysis of the causes of inbreeding depression (Hedrick et al., 2016) and development of a commercial, multi-species 60K SNP genotyping chip tagging 96% of the genome with 1 SNP every 12–20 kb (Silva-Junior et al., 2015). The EuCHIP60K has been a key resource to investigate genome-wide recombination, linkage disequilibrium, and nucleotide diversity (Silva-Junior and Grattapaglia, 2015), carry out genome-wide association (Resende et al., 2016; Muller et al., 2017) and drive genomic selection in different *Eucalyptus* species (Duran et al., 2017; Resende et al., 2017; Tan et al., 2017, 2018). *Eucalyptus* transcriptomics resources are available at https://eucgenie.org/. Ongoing and future efforts is focusing on understanding genome diversity and evolution in this species-rich genus and other members of the Myrtaceae (Grattapaglia et al., 2012), including sister genera such as *Corymbia* for which a genome assembly, soon to be released, revealed conservation but dynamic evolution of terpene genes relative to *Eucalyptus* (Butler et al., 2018).

### Salix

Whole-genome analysis in willow (*Salix* spp.), including development of reference genome assemblies, have focused on four species: *Salix purpurea* L. (Section Helix), *S. suchowensis* Cheng Section Helix), *S. wilsonii* Seemen (Section Wilsonianae) and *S. viminalis* L. (Section Vimen). The genome of a female *S. purpurea* ‘94006’ was assembled at the J. Craig Venter Institute from Illumina sequence generated at the DOE Joint Genome Institute, including mate-pair library reads, and scaffolds were ordered using a genetic map generated from an F_2_ mapping population genotyped using genotyping-by-sequencing at Cornell University^1^, placing ∼70% of the genome (276 Mb) in chromosome-scale pseudomolecules. The *S. suchowensis* genome was sequenced using a combination of Roche 454 technology and Illumina/HiSeq 2000 reads (Dai et al., 2014). Additional genome sequencing projects are currently underway in *S. purpurea*, *S. wilsonii*, and *S. viminalis* using deep PacBio sequencing, which should result in much more contiguous and complete genomes. Sequence information and gene assemblies for multiple *Salix* species are now available at TreeGenes^5^.

The genome sequencing projects have revealed three notable differences between the *Populus* and *Salix* genomes. First, the *Salix* genomes are clearly smaller than the *Populus* genomes. Using 17-mer analysis, the *S. suchowensis* genome size was estimated at ∼425 Mb (Dai et al., 2014), while the *S. purpurea* genome was estimated to be ∼379 Mb by 25-mer analysis, both of which are slightly smaller than the estimate of ∼429 and 450 Mb from flow cytometry of each species, respectively. Notwithstanding, all of these estimates are smaller than the genome size of *P. trichocarpa* (∼485 Mb). The *Salix* genomes also show evidence of more extensive fractionation following the whole-genome Salicoid duplication that is shared by *Salix* and *Populus* (Tuskan et al., 2006), resulting in a reduced number of predicted genes and overall smaller genome size (Dai et al., 2014). Second, it is clear that the large chromosome 1 in *Populus* corresponds to portions of chromosome 1 and chromosome 16 in *Salix*, suggesting a series of major chromosomal fission and fusion events occurred after the Salicoid duplication leading to the divergence of these lineages (Berlin et al., 2010; Hou et al., 2016). Finally, the sex determination locus is located on chromosome 15 in *Salix*, while it is on chromosome 19 in all *Populus* species studied to date, supporting the hypothesis that there has been greater genome fractionation in *Salix* following the Salicoid duplication event. Furthermore, most *Populus* species have an XY sex determination system (Tuskan et al., 2012; Geraldes et al., 2015), while all sequenced *Salix* species have a ZW system (Hou et al., 2015; Pucholt et al., 2015; Chen et al., 2016).

Given the apparently dynamic nature of sex determination in this dioecious family, rigorous efforts to understand this trait have been undertaken in recent years. Extensive differential gene expression has been documented in shoot tips of *S. purpurea*, which display an abundance of female-biased gene expression (Carlson et al., 2017). Expression analysis in *S. viminalis* suggests a predominance of male-biased gene expression, perhaps due to masculinization of the Z chromosome (Pucholt et al., 2017). Analysis of differentially expressed genes in unflushed buds of *S. suchowensis* identified 806 differentially expressed genes between males and females (Liu et al., 2015).

There are also extensive genetic mapping resources to support efforts to relate genotype to phenotype and accelerate willow breeding using molecular tools. In addition to the F_2_ map used to anchor the *S. purpurea* genome assemblies (Kopp et al., 2002), more than a dozen linkage mapping populations have been developed for *S. viminalis* (Karp et al., 2011), as well as an association mapping population (Hallingbäck et al., 2016). New mapping populations have also been developed based on an approach of using *S. purpurea* male ‘94001’ and female ‘94006’ reference individuals as common parents crossed with a number of other species, including *S. viminalis*, *S. suchowensis*, *S. integra*, *S. koriyanagi*, and *S. udensis* at Cornell University. At Nanjing Forestry University, an effort has been employed to establish recombinant inbred lines for *S. suchowensis*, with an inbred F_4_ pedigree currently established in the field.

### Quercus

A highly contiguous haploid genome of a heterozygous pedunculate oak (*Quercus robur*) was recently generated based on Illumina synthetic long-reads and 454 (Roche) sequences (Plomion et al., 2018). The assembly contains 1,409 scaffolds totaling 814 Mb (N50, 1.34 Mb)^6^. Overall 871 scaffolds, representing 96% of the physical size of the genome, were anchored to the 12 chromosomes. Based on RNA-seq and protein homologies, 25,808 genes were predicted. Two other draft genomes, while of lower quality, have also been published (Sork et al., 2016; Schmid-Siegert et al., 2017). As in *Eucalyptus*, one of the main features of the oak genome is its remarkably high level of proximal tandem duplication (35.6% of the gene models). The tight relationships between duplicated genes and lineage-specific selection already reported in other species was found to be particularly exacerbated in oak, as three quarters of the genes in expanded orthogroups were also found in tandem duplications. Another interesting feature of long-lived tree species rests in the accumulation of somatic mutations. The presence and transmission of somatic mutations was recently demonstrated in oak (Schmid-Siegert et al., 2017; Plomion et al., 2018), which raises interesting questions related to the role of somatic mutation in the evolution of long-lived species with a potentially high mutational load.

One of the major challenges in plant biology is identifying and characterizing the genes responsible for variation in ecologically and economically important traits. Genetic architecture of traits related to growth, phenology, water metabolism, and defense against pathogens has been explored in different full-sib oak pedigrees (e.g., Brendel et al., 2008) and natural populations (e.g., Alberto et al., 2013). Linkage and association mapping revealed many underlying loci with low to moderate contributions to the trait of interest. The characterization of the oak transcriptome is more recent. Gene repertoires were first constructed (Ueno et al., 2010; Pereira-Leal et al., 2014; Cokus et al., 2015; Lesur et al., 2015) and RNA-seq data were then used to (1) analyze molecular plasticity under abiotic stress (e.g., drought, Spieß et al., 2012) and biotic interactions (e.g., with insects, Kersten et al., 2013) and (2) identify genes and gene networks underlying developmental mechanisms (e.g., bud phenology, Ueno et al., 2013 and acorn development, Miguel et al., 2015) and evolutionary processes including reproductive isolation (Le Provost et al., 2012, 2016) and local adaptation (Gugger et al., 2016a). Recent studies provide evidence for epigenetic variation in phenotypic plasticity and evolutionary processes in oaks (Gugger et al., 2016b), establishing new avenues for research into the role of epigenetics in trait plasticity for these long-lived species.

Shortlisted among the ‘botanical horror stories’ (Rieseberg et al., 2006), the *Quercus* genus constitutes an ideal taxon to investigate the dynamics of lineage diversification along a wide and fluid continuum of speciation. Several efforts to illuminate oak evolutionary histories using population genomic approaches have recently emerged (Ortego et al., 2016, 2018; Leroy et al., 2017, 2018). In European white oaks for example, the best-supported scenario of divergence is consistent with a long-term pervasive effect of interspecific gene flow, with the exception of some narrow genomic regions responsible for reproductive isolation. Further work will be required to identify genes targeted by intrinsic or ecological selection, but early attempts appear promising and could enable predictions of the evolutionary responses of oaks to climate change (Gugger et al., 2016a; Rellstab et al., 2016). An exciting perspective in population genomics is presented by allochronic approaches that compare ancient and modern DNA to derive evolutionary changes at targeted genomic regions. Wagner et al. (2018) have suggested ancient DNA could be retrieved from waterlogged archeological or fossil samples, thus enabling the incremental reconstruction of evolutionary trajectories.

## Chestnut, Pecan, and Other Early Draft Angiosperm Genomes

Other reference genome projects for hardwoods are underway, including efforts to produce reference sequences for chestnut and pecans. The chestnut project aims to produce reference genomes of both *Castanea mollissima* (Chinese chestnut), the donor of resistance to *Cryphonectria parasitica* (chestnut blight) and *Castanea dentata* (American chestnut) the species destroyed by the blight in the early 1900s. The Forest Health Initiative (Nelson et al., 2014) supported a project to sequence the genome of the Chinese chestnut donor parent tree ‘Vanuxem’ used by The American Chestnut Foundation’s (TACF). The first draft of the Chinese chestnut genome assembly was released in January 2014 at: https://www.hardwoodgenomics.org/chinese-chestnut-genome, consisting of 41,260 scaffolds averaging 39.6 kb in length and covering 98% (∼724 Mb) of the genome. Targeted regions of mapped QTL resistance loci from various clones have also been produced (Staton et al., 2015). In addition, work is underway to prepare chromosome-length sequences for the Chinese chestnut genome. Long, single-molecule PacBio data are being used to merge contigs, followed by anchoring of scaffolds to high-density linkage maps for the cv. Vanuxem. Fluorescent *in situ* hybridization is being used to validate the chromosome-level assemblies and subsequently to identify structural rearrangements among genotypes and species (Islam-Faridi et al., 2016). The Chinese chestnut reference genome is being utilized to develop genomic selection models for blight resistance in American chestnut backcross populations (Westbrook, 2018). The project to sequence the American chestnut has produced a contig assembly, based on PacBio sequencing, of 8.1 Mb contig N50 and efforts are now underway to use new technologies (see below) and genetic mapping to produce chromosome-scale assemblies for other *C*. *mollissima* and *C*. *dentata* genotypes.

Initial efforts at generating a *Carya illinoinensis* (pecan) reference genome, released in 2013 and based on Illumina short-read sequencing (Jenkins et al., 2015) resulted in a contig N50 of 6.5 kb and scaffold N50 of 11.2 kb. The Mexican accession ‘87MX3-2.11’ from the USDA-ARS facility in Somerville, Texas was selected as the reference genome based on a reduced heterozygosity. Efforts are underway to generate PacBio-based genomes for pecans that include 87MX3-2 and three production cultivars^7^. The current version of the pre-chromosome assembly for 87MX3-2 covers 705.6 Mb of the genome with a contig N50 of 2.5 Mb with 36,489 genes annotated^1^. Multiple early efforts are also underway to sequenced the European ash (*Fraxinus excelsior*) and American ash (*F. americana*)^8^^,^^9^, as well as several birch genomes (*Betula nana*, *B*. *pubescens*, and *B. pendula*) (Wang et al., 2013). Finally, several angiosperm genomes are in early draft form and include species of ash^5^ and walnut^9^, as well as several comparative angiosperms from horticultural species^10^.

## A Look to the Near Future

The continued development of new technologies that can be applied to generate high-quality hardwood reference genomes has been fostered by single-molecule, real-time sequencing (SMRT) and related assembly algorithms from PacBio (Eid et al., 2009). These long reads, which average 10–15 kb in length, but are frequently 20–50 kb in length, can be used to resolve complex genomic repeats and improve contiguity of genomes. An early example of a small, inbred, grass genome (*Oropetium thomaeum*) achieved a contig N50 of 2.4 Mb, while capturing almost the entire genome space (244 out of 245 Mb) (VanBuren et al., 2015). The PacBio long reads were also used to produce a reference for the large, complex sunflower (*Helianthus annuus*) genome (Badouin et al., 2017) at 3.6 Gb with a contig N50 of 524 kb. An alternative reference to the *Zea mays* B73 supports using PacBio Iso-Seq sequencing for collecting full-length cDNAs to enhance the reference annotation and a genome (Jiao et al., 2017), which despite large-scale nested transposon activity, was completed at a contig N50 of 1.18 Mb. Because of the low accuracy rate of individual PacBio reads, these reads must be combined with an alternative technology, such as Illumina short reads, to polish the consensus sequence. For hardwood tree genomes, the longer reads facilitate the generation of outbred reference sequences to sort haplotypes and produce a single reference despite high heterozygosity rates (see *Q. robur*, *P. deltoides* WV94 and *Castanea dentata* described above). In addition to long-read sequencing, which has brought us back to the completeness of Sanger-based sequencing, new tools are emerging to achieve accurate chromosome-scale contiguity. These tools for genomic mapping and assembly include improved single-molecule mapping from BioNano (Staňková et al., 2016), *in vitro* reconstituted chromatin (Putnam et al., 2016) and binned sequencing approaches such as 10x genomics (Coombe et al., 2016). The most promising of these technologies is Hi-C for ordering large contigs from genome assemblies. Originally developed to perform “contact mapping” in human cell lines to show genes adjacent to promoter or regulatory elements (Suhas, 2014), it has been repurposed as a general solution to determining order in genome assemblies (Dudchenko et al., 2017). A recent example, the 4.8 Gb genome of *Hordeum vulgare*, has shown the ability to use Hi-C to precisely place 95% of contigs on pseudomolecules (Mascher et al., 2017). All of these tools are actively being applied to improve hardwood genomic references as a foundation for accurate population and functional analysis.

These new technologies will enable greater within and across species comparisons. An example of unique tree biology, that can be explored, is the characterization of somatic mutations. Due to their perennial habit, trees can accumulate somatic mutations in alternate vegetative lineages (as noted in the *Populus* and *Quercus* sections above). However, the effect of somatic variation on the generation of new genetic variation at the population-level and/or during reproduction remains largely unknown. Since, in plants, germlines are not segregated from the vegetative lineages, somatic mutations can be transmitted to the next generation. The frequency with which a given somatic mutation propagates to the next generation depends both on the overall fitness of the tree and the probability of the somatic sector giving rise to flowers and gametes. Because branches/tissue types do not make equal contributions to the resulting gene pool, somatic mutations may alter the adaptive balance between branches. This mosaic genetic architecture raises the possibility that selection acts simultaneously on both the branch and tree (Hadany, 2001; Clarke, 2011). Understanding the role of multi-level selection within a single tree will require answering several questions. That is, to what extent is the effect of a somatic mutation in a single branch shared with the entire tree (Folse and Roughgarden, 2011)? And, if a branch acquires a broadly-beneficial mutation, such as one conferring resistance to herbivory (e.g., Edwards et al., 1990), does the resistant branch gain a larger fitness benefit than the remainder of the tree? Conversely, can a mutation increase the fitness of a single branch despite imposing cost on the rest of the tree? For example, a mutation that increases flowering in a branch might increase seed production from that branch while depressing production from the tree as a whole by acting as a metabolic sink (Walbot, 1985). Finally, should these conflicts occur, do trees have mechanisms to mediate resource allocation among branches, and, if so, are these mechanisms common between independent tree lineages? New techniques (described above) for finding or constructing whole-genome genetic mosaic and chimeric trees with phenotypically-relevant somatic mutations are now allowing these questions to be addressed.

In summary, the assembly and annotation of multiple hardwood tree genomes has facilitated an increase in (1) functional characterization of genes and gene networks related to tree habit, (2) GWAS and genomic selection investigations and applications, and (3) comparative genomic efforts among various tree species. The rapidly expanding new technologies will add even greater number of hardwood species to these efforts. The power of comparative genomics will increase our understanding of how these highly dynamic genomes have evolved and resulted in the amazing array of phenotypic diversity found among and within hardwood species. To broaden the raft of available hardwood genomes, resources should be directed toward additional candidate hardwood genera, including but is not limited to *Liriodendron*, *Liquidambar*, *Swietenia*, and *Acer*. In sum total, most hardwoods are undomesticated long-lived plants that provide many ecological and commercial benefits, whose management, conservation and domestication for economic and ecological purposes will benefit from a set of rich genomics resources. Ultimately such resources will favorably impact the pressing problems of climate change, soil and water conservation, bioenergy and biomaterials production, and maintenance of heathy ecosystem functions. As a result, we may finally answer the questions of: (1) why has the tree habit evolved repeatedly in the angiosperms and (2) what is the connection between the genomes and the defining characteristics of long-lived perennial plants?

## Author Contributions

All authors contributed equally to the formatting and writing contained in this Mini-review.

## Conflict of Interest Statement

The authors declare that the research was conducted in the absence of any commercial or financial relationships that could be construed as a potential conflict of interest.

## Footnotes

1 https://phytozome.jgi.doe.gov

2 https://jgi.doe.gov/doe-jgi-plant-flagship-gene-atlas/

3 https://cbi.ornl.gov/

4 http://popgenie.org/aspseq

5 https://www.treegenesdb.org/

6 http://www.oakgenome.fr/

7 pecantoolbox.nmsu.edu">http://pecantoolbox.nmsu.edu">pecantoolbox.nmsu.edu

8 http://www.ashgenome.org/

9 http://hardwoodgenomics.org

10 https://www.rosaceae.org/
